# Supplementary material for: The Evolution and Characterization of the RNA Interference Pathways in Lophotrochozoa
Source: Genome Biol Evol. 2024 May 7;16(5):evae098. doi: 10.1093/gbe/evae098 (PMC11114477; doi:10.1093/gbe/evae098)
Supplement: evae098_Supplementary_Data [file evae098_supplementary_data.zip › Supplementary_materials.zip!/selected_miRNAs/NoGe_selected_miRNAs/mirdeep_pdf_files/NMRB01001254.1_Notospermus_geniculatus_strain_Ushimado_scaffold1254,_whole_genome_shotgun_sequence_26722.pdf]

Provisional ID : NMRB01001254.1_Notospermus_geniculatus_strain_Ushimado_scaffold1254,_whole_genome_shotgun_sequence_26722
Score total : 30550.9
Score for star read(s) : 3.9
Score for read counts : 30542.7
Score for mfe : 1.3
Score for randfold : 0
Score for cons. seed : 3
Total read count : 59920
Mature read count : 59868
Loop read count : 0
Star read count : 52

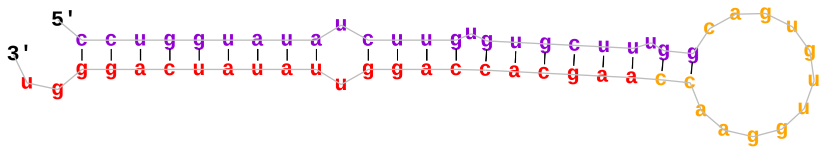

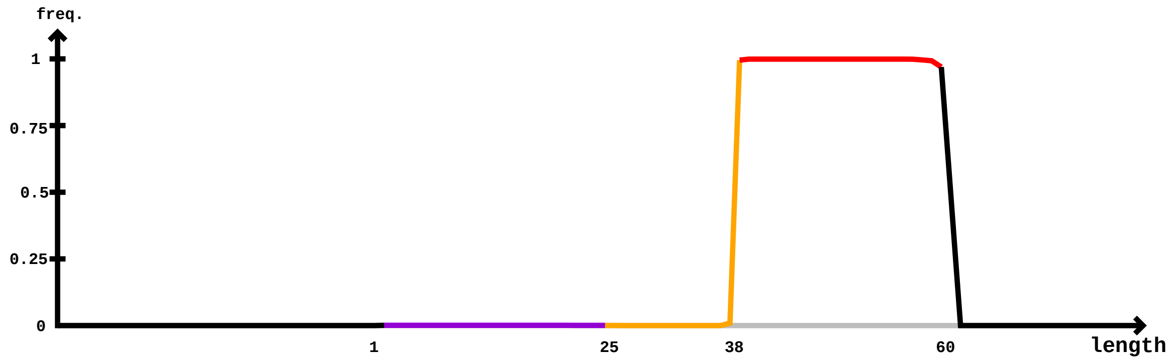


```
                                       Star                               Mature
5'- cguuauaaucgagcuuaaugccuuacuugaacccugguauaucuugugugcuuuggcaguguuggaaccaagcaccagguuauaucaggguguuugcaucgcauguacaaa  -3'  obs
    cguuauaaucgagcuuaaugccuuacuugaacccugguauaucuugugugcuuuggcaguguuggaaccaagcaccagguuauaucaggguguuugcaucgcauguacaaa       exp
    ........(((((.............)))))(((((((((((.((((.((((((.((..........)))))))))))).)))))))))))((.(((...))).)).....   reads   mm   sample
    .....................................................................aagcaccagguuauauca........................   8       0    Mm1
    .....................................................................aagcaccagguuauaucag.......................   1       0    Mm1
    .....................................................................aagcaccagguuauaucagg......................   4       0    Mm1
    .....................................................................aagcaccGgguuauaucaggg.....................   1       1    Mm1
    .....................................................................aagcaccagguuauauUaggg.....................   1       1    Mm1
    .....................................................................aagcaccagguuauaucGggg.....................   2       1    Mm1
    .....................................................................aagcaccagguuauaucaggg.....................   140     0    Mm1
    .....................................................................aagcaccagguuauaucGgggu....................   2       1    Mm1
    .....................................................................aagcaccagguuauaucagggu....................   1242    0    Mm1
    .....................................................................Gagcaccagguuauaucagggu....................   1       1    Mm1
    .....................................................................aagcaccagguuauauUagggu....................   4       1    Mm1
    .....................................................................aagcacUagguuauaucagggu....................   1       1    Mm1
    .....................................................................Nagcaccagguuauaucagggu....................   1       1    Mm1
    .....................................................................aagcaccaggAuauaucagggu....................   4       1    Mm1
    .....................................................................Uagcaccagguuauaucagggu....................   1       1    Mm1
    .....................................................................aagcaccagguuauauAagggu....................   5       1    Mm1
    .....................................................................aagcaccaggGuauaucagggu....................   2       1    Mm1
    .....................................................................aaUcaccagguuauaucagggu....................   2       1    Mm1
    .....................................................................aagcaccagguuauaucagggG....................   1       1    Mm1
    .....................................................................aagcaccagguuauaucUgggu....................   4       1    Mm1
    .....................................................................aagcaccagguuauauGagggu....................   3       1    Mm1
    .....................................................................aagcaccagguuauaucCgggu....................   6       1    Mm1
    .....................................................................aagAaccagguuauaucagggu....................   1       1    Mm1
    .....................................................................aagcaccagguuauGucagggu....................   1       1    Mm1
    .....................................................................aagcaccagguuauaucagggC....................   74      1    Mm1
    .....................................................................aagcGccagguuauaucagggu....................   1       1    Mm1
    .....................................................................aagcaccagguuauaucaggguU...................   13      1    Mm1
    .....................................................................aagcaccagguuauaucaggguA...................   35      1    Mm1
    ......................................................................agcaccagguuauaucagggu....................   5       0    Mm1

    .............cuuaaugccuuacuugaac...............................................................................   1       0    Mm2
    ................................ccugguauaucuugugugcu...........................................................   2       0    Mm2
    ................................ccugguauaucuugugugcuuu.........................................................   2       0    Mm2
```

```
                                Star                                Mature
cguuauaaucgagcuuaaugccuuacuugaacccugguauaucuugugugcuuuggcaguguuggaaccaagcaccagguuauaucaggguggcuugcaucgcauguacaaa
................................ccugguauaucuugugugcuuug........................................................    1      0     Mm2
................................ccugguauaucuugugugcuuugg.......................................................    14     0     Mm2
........................................Uucuugugugcuuuggca.....................................................    1      1     Mm2
...................................................................Ucaagcaccagguuauaucagggu....................    2      1     Mm2
....................................................................caagcaccagguuauauAaggg.....................    1      1     Mm2
....................................................................caagcaccagguuauaucaggg.....................    6      0     Mm2
....................................................................caagcaccagAuuauaucagggu....................    1      1     Mm2
....................................................................caagcacAagguuauaucagggu....................    1      1     Mm2
....................................................................caagcaccagguuauaucagggu....................    6      0     Mm2
....................................................................caagcaccagguuauaucagggC...................    1      1     Mm2
.....................................................................aagcaccagguuauauca........................    2      0     Mm2
.....................................................................aagcaccagguuauaucaA.......................    1      1     Mm2
.....................................................................aagcaccagguuauaucag.......................    7      0     Mm2
.....................................................................aagcaccagguuauaucGg.......................    1      1     Mm2
.....................................................................aagcaccagguuauaucUgg......................    1      1     Mm2
.....................................................................aagcaccGgguuauaucagg......................    1      1     Mm2
.....................................................................aagcaccagguuauaucagg......................    14     0     Mm2
.....................................................................aagcaccagguuauauAaggg.....................    1      1     Mm2
.....................................................................aagcaccagguuauaucaggA.....................    1      1     Mm2
.....................................................................aagcaccagguuGuaucaggg.....................    1      1     Mm2
.....................................................................aagcaccagguuauaucUggg.....................    2      1     Mm2
.....................................................................aagcaccagguuauauUaggg.....................    3      1     Mm2
.....................................................................aagcaccaggAuauaucaggg.....................    1      1     Mm2
.....................................................................aagcaccagguuauaucCggg.....................    1      1     Mm2
.....................................................................aagcaccagguuauaCcaggg.....................    1      1     Mm2
.....................................................................aaAcaccagguuauaucaggg.....................    2      1     Mm2
.....................................................................aagcaccagguuauaucGggg.....................    2      1     Mm2
.....................................................................aagcaccagguuauaucaggU.....................    1      1     Mm2
.....................................................................aagcaccGgguuauaucaggg.....................    4      1     Mm2
.....................................................................aagcaccagguuauauGaggg.....................    1      1     Mm2
.....................................................................aagcaccagguuauaucaggg.....................    348    0     Mm2
.....................................................................aagcaccagguuaAaucagggu....................    2      1     Mm2
.....................................................................aagcacAagguuauaucagggu....................    1      1     Mm2
.....................................................................aaAcaccagguuauaucagggu....................    6      1     Mm2
.....................................................................aagcaccagguuaGaucagggu....................    1      1     Mm2
.....................................................................aagcaccagguuauaucagggC....................    302    1     Mm2
.....................................................................aagcaccaUguuauaucagggu....................    2      1     Mm2
.....................................................................aagcaccagguuauaucaAggu....................    1      1     Mm2
.....................................................................aagAaccagguuauaucagggu....................    5      1     Mm2
.....................................................................aagcaccagguuUuaucagggu....................    4      1     Mm2
.....................................................................aagcaccagguuauaAcagggu....................    1      1     Mm2
.....................................................................aagcaccagguuauaucUgggu....................    75     1     Mm2
.....................................................................aagcaccagguuauaucagAgu....................    2      1     Mm2
.....................................................................aagcaccagguuauauUagggu....................    52     1     Mm2
.....................................................................aagcaccagguuauaCcagggu....................    3      1     Mm2
.....................................................................aagcaccagguuGuaucagggu....................    1      1     Mm2
.....................................................................aagcaccagguuaCaucagggu....................    2      1     Mm2
.....................................................................aagcaccagguCauaucagggu....................    4      1     Mm2
.....................................................................aagcaccagguGauaucagggu....................    2      1     Mm2
.....................................................................Uagcaccagguuauaucagggu....................    16     1     Mm2
.....................................................................aagcaccagguuauaucagUgu....................    1      1     Mm2
.....................................................................aagcCccagguuauaucagggu....................    2      1     Mm2
.....................................................................aagcaccCgguuauaucagggu....................    1      1     Mm2
.....................................................................aagcaccagAuuauaucagggu....................    2      1     Mm2
.....................................................................aagcaccagguuauauGagggu....................    23     1     Mm2
.....................................................................aGgcaccagguuauaucagggu....................    7      1     Mm2
.....................................................................aagcaccaggAuauaucagggu....................    43     1     Mm2
.....................................................................aagcaccagguuauaucaggAu....................    1      1     Mm2
.....................................................................aagcaccagguuauaucGgggu....................    78     1     Mm2
.....................................................................aagcaccGgguuauaucagggu....................    76     1     Mm2
.....................................................................aagcaccagguuauaucagggu....................    20898  0     Mm2
.....................................................................aagcaccagUuuauaucagggu....................    1      1     Mm2
.....................................................................Gagcaccagguuauaucagggu....................    2      1     Mm2
.....................................................................aagcaUcagguuauaucagggu....................    2      1     Mm2
.....................................................................aagcaccagguuauaucagCgu....................    1      1     Mm2
.....................................................................aagcaccagguuauaucaUggu....................    1      1     Mm2
.....................................................................aagcaccUgguuauaucagggu....................    4      1     Mm2
.....................................................................aagcGccagguuauaucagggu....................    2      1     Mm2
.....................................................................aagcaccagguuauUucagggu....................    3      1     Mm2
.....................................................................Nagcaccagguuauaucagggu....................    18     1     Mm2
```

Star — Mature

```
cguuauaaucgagcuuaaugccuuacuugaacccugguauaucuugugugcuuuggcaguguuggaaccaagcaccagguuauaucagggugcuugcaucgcauguacaaa
.....................................................................aagcaccaggGuauaucagggu....................	22	1	Mm2
.....................................................................aagcaccagguuauaucagggA....................	10	1	Mm2
.....................................................................aaUcaccagguuauaucagggu....................	4	1	Mm2
.....................................................................aagcaGcagguuauaucagggu....................	1	1	Mm2
.....................................................................aagcaccaggCuauaucagggu....................	23	1	Mm2
.....................................................................aagcaccagguuauaucaggCu....................	6	1	Mm2
.....................................................................aUgcaccagguuauaucagggu....................	1	1	Mm2
.....................................................................aagcaccagguuauaucCgggu....................	80	1	Mm2
.....................................................................aagcaccagguuauauAagggu....................	82	1	Mm2
.....................................................................aagcaccagguuauaucagggG....................	330	1	Mm2
.....................................................................aaCcaccagguuauaucagggu....................	1	1	Mm2
.....................................................................aagcacGagguuauaucagggu....................	1	1	Mm2
.....................................................................aagcaccagCuuauaucagggu....................	1	1	Mm2
.....................................................................aCgcaccagguuauaucagggu....................	2	1	Mm2
.....................................................................aagcaNcagguuauaucagggu....................	4	1	Mm2
.....................................................................aagcacNagguuauaucagggu....................	2	1	Mm2
.....................................................................aagGaccagguuauaucagggu....................	1	1	Mm2
.....................................................................aagcacUagguuauaucagggu....................	12	1	Mm2
.....................................................................aagcaccagguuauGucagggu....................	3	1	Mm2
.....................................................................aagcaccagguuauaucagggug...................	1	0	Mm2
.....................................................................aagcaccagguuauaucaggguA...................	178	1	Mm2
.....................................................................aagcaccagguuauaucaggguU...................	569	1	Mm2
......................................................................agcaccagguuauaucaggg.....................	1	0	Mm2
......................................................................agcaccagguuauaucagggG....................	2	1	Mm2
......................................................................agcaccaCguuauaucagggu....................	1	1	Mm2
......................................................................agcaccaggAuauaucagggu....................	1	1	Mm2
......................................................................agcaccagguuauaucCgggu....................	1	1	Mm2
......................................................................agcaccagguuauaucagggu....................	67	0	Mm2
......................................................................agcaccagguuauaucagggC....................	1	1	Mm2
......................................................................agcaccagguuauaucaggguC...................	1	1	Mm2
......................................................................agcaccagguuauaucaggguU...................	1	1	Mm2
.......................................................................gcaccagguuauaucagggu....................	1	0	Mm2

................................ccugguauaucuugugugcu...........................................................	5	0	Fm1
.....................................................................aagcaccagguuauauca........................	2	0	Fm1
.....................................................................aagcaccagguuauaucaU.......................	1	1	Fm1
.....................................................................aagcaccagguuauaucag.......................	2	0	Fm1
.....................................................................aagcGccagguuauaucag.......................	1	1	Fm1
.....................................................................aagcaccGgguuauaucag.......................	1	1	Fm1
.....................................................................aagcaccagguuauaucagg......................	5	0	Fm1
.....................................................................aagcaccaggAuauaucaggg.....................	1	1	Fm1
.....................................................................aagcaccagguuauaucGggg.....................	1	1	Fm1
.....................................................................aagcaccagguuauauAaggg.....................	1	1	Fm1
.....................................................................aagcaccagguuauaucaggg.....................	121	0	Fm1
.....................................................................aGgcaccagguuauaucagggu....................	4	1	Fm1
.....................................................................aaAcaccagguuauaucagggu....................	3	1	Fm1
.....................................................................aaCcaccagguuauaucagggu....................	1	1	Fm1
.....................................................................aagcaccagguuauauUagggu....................	22	1	Fm1
.....................................................................aagcaccagguuauaucagggu....................	9116	0	Fm1
.....................................................................aagcaccCgguuauaucagggu....................	1	1	Fm1
.....................................................................aagcaccagguuauaucaggUu....................	2	1	Fm1
.....................................................................aagcacAagguuauaucagggu....................	1	1	Fm1
.....................................................................aagcaccagguuauGucagggu....................	1	1	Fm1
.....................................................................aagcaccagguuauauGagggu....................	10	1	Fm1
.....................................................................aagcaccagguuauUucagggu....................	2	1	Fm1
.....................................................................aaUcaccagguuauaucagggu....................	1	1	Fm1
.....................................................................aagcaccagguuauauAagggu....................	39	1	Fm1
.....................................................................aagcaccagguuauaAcagggu....................	1	1	Fm1
.....................................................................aagcaccagguuaAaucagggu....................	1	1	Fm1
.....................................................................aagcaccagguuauaucaUggu....................	3	1	Fm1
.....................................................................aagcaccagguuauaucagggG....................	175	1	Fm1
.....................................................................aagcaccaggGuauaucagggu....................	7	1	Fm1
.....................................................................aagcaccagguuauaucaggCu....................	1	1	Fm1
.....................................................................aagcaccagguuauaucagUgu....................	2	1	Fm1
.....................................................................aagcaccagguuauaucUgggu....................	37	1	Fm1
.....................................................................Uagcaccagguuauaucagggu....................	7	1	Fm1
.....................................................................aNgcaccagguuauaucagggu....................	1	1	Fm1
.....................................................................Nagcaccagguuauaucagggu....................	2	1	Fm1
.....................................................................aagcaGcagguuauaucagggu....................	1	1	Fm1
.....................................................................aagcUccagguuauaucagggu....................	2	1	Fm1
```

Star Mature

```
cguuauaaucgagcuuaaugccuuacuugaacccugguauaucuugugugcuuuggcaguguuggaaccaagcaccagguuauaucaggguGcuugcaucgcauguacaaa
.....................................................................aagUaccagguuauaucagggu....................   2      1   Fm1
.....................................................................aagcaccagguuauaucagAgu....................   1      1   Fm1
.....................................................................aagcaccagguuGuaucagggu....................   2      1   Fm1
.....................................................................aagcaccagguuauaucagggC....................   115    1   Fm1
.....................................................................Gagcaccagguuauaucagggu....................   4      1   Fm1
.....................................................................aagcaccGgguuauaucagggu....................   59     1   Fm1
.....................................................................aagcaccagguuauaucCgggu....................   27     1   Fm1
.....................................................................aagcacUagguuauaucagggu....................   6      1   Fm1
.....................................................................aagcaccagguuauaucagggA....................   5      1   Fm1
.....................................................................aagcaccagguuauaCcagggu....................   1      1   Fm1
.....................................................................aagcaccagguuauaucaAggu....................   3      1   Fm1
.....................................................................aagcaccagUuuauaucagggu....................   1      1   Fm1
.....................................................................aagcaccaggAuauaucagggu....................   13     1   Fm1
.....................................................................aagcaccagguuauaucaggAu....................   2      1   Fm1
.....................................................................aagcaNcagguuauaucagggu....................   2      1   Fm1
.....................................................................aagcaccagguuauaucGgggu....................   37     1   Fm1
.....................................................................aagcaccaggCuauaucagggu....................   10     1   Fm1
.....................................................................aagcaccUgguuauaucagggu....................   2      1   Fm1
.....................................................................aagcaccagguuauaucaggguA...................   97     1   Fm1
.....................................................................aagcaccagguuauaucaggguU...................   589    1   Fm1
......................................................................agcaccagguuauaucaggg.....................   2      0   Fm1
......................................................................agcaccagguAauaucagggu....................   1      1   Fm1
......................................................................agcaccagguuauaucagggu....................   28     0   Fm1
......................................................................agcaccagguuauaucaggUu....................   1      1   Fm1

.....................................................................aagcaccagguuaAaucagg......................   1      1   Fm2
.....................................................................aagcaccagguuauaucagg......................   20     0   Fm2
.....................................................................aagcaccagguuauaucaggg.....................   48     0   Fm2
.....................................................................aagcaccGgguuauaucagggu....................   10     1   Fm2
.....................................................................aagcaccagguuauaucCgggu....................   7      1   Fm2
.....................................................................aagcaccagguuauaucagggG....................   47     1   Fm2
.....................................................................aagcaccagguuauauGagggu....................   4      1   Fm2
.....................................................................aaUcaccagguuauaucagggu....................   3      1   Fm2
.....................................................................aagcaccagguuauauAagggu....................   8      1   Fm2
.....................................................................aagcaccaggAuauaucagggu....................   5      1   Fm2
.....................................................................aagcaccagguuauaucaggUu....................   1      1   Fm2
.....................................................................aagcaUcagguuauaucagggu....................   1      1   Fm2
.....................................................................aagcaccaggCuauaucagggu....................   5      1   Fm2
.....................................................................aagcaccagguuGuaucagggu....................   2      1   Fm2
.....................................................................aagcaccagguuauaucUgggu....................   10     1   Fm2
.....................................................................Gagcaccagguuauaucagggu....................   1      1   Fm2
.....................................................................aagcaccagguuauaucGgggu....................   9      1   Fm2
.....................................................................aagcaccagguuaAaucagggu....................   2      1   Fm2
.....................................................................aagcaccagguuauaucagAgu....................   1      1   Fm2
.....................................................................aagcacUagguuauaucagggu....................   1      1   Fm2
.....................................................................Uagcaccagguuauaucagggu....................   1      1   Fm2
.....................................................................aagcaccagguuauaucagUgu....................   2      1   Fm2
.....................................................................aagAaccagguuauaucagggu....................   1      1   Fm2
.....................................................................aagcaccagguuauaucagggu....................   2526   0   Fm2
.....................................................................aGgcaccagguuauaucagggu....................   9      1   Fm2
.....................................................................aagcaccaggGuauaucagggu....................   2      1   Fm2
.....................................................................aagcaccagguuauaucaggAu....................   1      1   Fm2
.....................................................................aagcaccagguuauaucagggA....................   1      1   Fm2
.....................................................................aagcaccagguuauaucagggC....................   27     1   Fm2
.....................................................................aagcaccagguuauauUagggu....................   6      1   Fm2
.....................................................................aagcaccagguuauaucaggguA...................   37     1   Fm2
.....................................................................aagcaccagguuauaucagggug...................   1      0   Fm2
.....................................................................aagcaccagguuauaucaggguU...................   126    1   Fm2

................................ccugguauaucuugugugcu...........................................................   1      0   Mm3
................................ccugguauaucuugugugcuu..........................................................   1      0   Mm3
................................ccugguauaucuugugugcuuug........................................................   2      0   Mm3
................................ccugguauaucuugugugcuuugg.......................................................   13     0   Mm3
....................................................................caagcaccagguuauaucaggg.....................   1      0   Mm3
....................................................................caagcaccagguuauaucagggu....................   1      0   Mm3
.....................................................................aagcaccagguuauaucag.......................   3      0   Mm3
.....................................................................aagcaccagguuauaucagg......................   41     0   Mm3
.....................................................................aagcaccagguuauaucCgg......................   1      1   Mm3
.....................................................................aagcaccagguuauaucaggg.....................   205    0   Mm3
.....................................................................aagcGccagguuauaucaggg.....................   1      1   Mm3
```

Star **Mature**

cguuauaaucgagcuuaaugccuuacuugaacccugguauaucuugugugcuuuggcaguguuggaaccaagcaccagguuauaucaggguggcuugcaucgcauguacaaa			
....aagcaccaggCuauaucaggg.....................	1	1	Mm3
....aagcaccagguuauaucaggA.....................	1	1	Mm3
....aagcaccGgguuauaucaggg.....................	1	1	Mm3
....aUgcaccagguuauaucagggu....................	1	1	Mm3
....aagcaccagguuauaucUgggu....................	18	1	Mm3
....aagcacNagguuauaucagggu....................	1	1	Mm3
....aagcaccagguuauauUagggu....................	15	1	Mm3
....aagcaccaUguuauaucagggu....................	1	1	Mm3
....aagcaccagguuauaucGgggu....................	14	1	Mm3
....aCgcaccagguuauaucagggu....................	1	1	Mm3
....aagcUccagguuauaucagggu....................	1	1	Mm3
....aagcaccagguuauUucagggu....................	1	1	Mm3
....aagcaccagguCauaucagggu....................	1	1	Mm3
....Uagcaccagguuauaucagggu....................	3	1	Mm3
....aagcaccagguuauGucagggu....................	2	1	Mm3
....aagcacGagguuauaucagggu....................	1	1	Mm3
....aagcaccagguuauauGagggu....................	4	1	Mm3
....aagAaccagguuauaucagggu....................	3	1	Mm3
....aagcaccagUuuauaucagggu....................	2	1	Mm3
....aagcGccagguuauaucagggu....................	1	1	Mm3
....aagcaccagguuauaucaAggu....................	2	1	Mm3
....aagcacUagguuauaucagggu....................	2	1	Mm3
....aagcaccUgguuauaucagggu....................	2	1	Mm3
....aagcaccaggGuauaucagggu....................	3	1	Mm3
....aagcaccagguuauauAagggu....................	17	1	Mm3
....aaUcaccagguuauaucagggu....................	3	1	Mm3
....aagcaccaggAuauaucagggu....................	11	1	Mm3
....aagcaccagguuauaucagggC....................	144	1	Mm3
....aagcaccagguuauaucagggG....................	45	1	Mm3
....aagcaccagguuGuaucagggu....................	3	1	Mm3
....aagGaccagguuauaucagggu....................	1	1	Mm3
....aagcaccagguuCuaucagggu....................	1	1	Mm3
....aGgcaccagguuauaucagggu....................	4	1	Mm3
....aagcaccagguuauaucaggCu....................	1	1	Mm3
....aagcaccagguuauaucagggA....................	5	1	Mm3
....aagcaccGgguuauaucagggu....................	16	1	Mm3
....aagcaccagguuauaucCgggu....................	23	1	Mm3
....aagcaccagAuuauaucagggu....................	1	1	Mm3
....Gagcaccagguuauaucagggu....................	1	1	Mm3
....aagcaUcagguuauaucagggu....................	2	1	Mm3
....aagcaccagguuauaucagUgu....................	3	1	Mm3
....aagcaccaggCuauaucagggu....................	7	1	Mm3
....aaAcaccagguuauaucagggu....................	1	1	Mm3
....aagcaccagguuauaucaggUu....................	2	1	Mm3
....aagcaccagguuauaAcagggu....................	1	1	Mm3
....aagcaccagguuaAaucagggu....................	1	1	Mm3
....Nagcaccagguuauaucagggu....................	1	1	Mm3
....aagcaccagguuauaucagggu....................	5061	0	Mm3
....aagcaccagguuauaucaggguA...................	54	1	Mm3
....aagcaccagguuauaucaggguU...................	157	1	Mm3
....aagcaccagguuauaucaggguUc..................	1	1	Mm3
....agcaccaggGuauaucagggu....................	1	1	Mm3
....agcaccagguuauaucagggC....................	4	1	Mm3
....agcaccagguuauaucagggu....................	12	0	Mm3
....agcaccagguuauaucagggA....................	1	1	Mm3
................................ccugguauaucuugugugcu...	6	0	Fm3
................................ccugguauaucuugugugcuuug..	1	0	Fm3
................................ccugguauaucuugugugcuuuggA..	3	1	Fm3
....caagcaccagguuauauca........................	1	0	Fm3
....caagcaccagguuauaucag.......................	3	0	Fm3
....caagcaccagguuauauAagg......................	1	1	Fm3
....caagcaccagguuauaucagg......................	21	0	Fm3
....caagcaccagguuauauGaggg.....................	1	1	Fm3
....caagcaccagguuauaucaggg.....................	34	0	Fm3
....caagcaccagguuauauAagggu....................	1	1	Fm3
....caagcaccagguuauauGagggu....................	2	1	Fm3
....caagcaccagAuuauaucagggu....................	1	1	Fm3
....caagcaccagguuauaAcagggu....................	1	1	Fm3
....caagcaccagguuauaCcagggu....................	1	1	Fm3

Star Mature

```
cguuauaaucgagcuuaaugccuuacuugaacccugguauaucuugugugcuuuggcaguguuggaaccaagcaccagguuauaucagggugcuugcaucgcauguacaaa
....................................................................caagcacUagguuauaucagggu....................   1      1    Fm3
....................................................................caagcaccagguuauauUagggu....................   3      1    Fm3
....................................................................caagcaccagguuauaGcagggu....................   1      1    Fm3
....................................................................caagcaccagguuauaucaUggu....................   1      1    Fm3
....................................................................caagcaccagguuauaucagggu....................   423    0    Fm3
....................................................................caagcaccagguuauaucagggC....................   14     1    Fm3
....................................................................caagcaccagCuuauaucagggu....................   2      1    Fm3
....................................................................Uaagcaccagguuauaucagggu....................   1      1    Fm3
....................................................................caagcaccagUuuauaucagggu....................   1      1    Fm3
....................................................................caagcaccagguuauaucaggguU...................   6      1    Fm3
.....................................................................aagcaccagguuauauAa........................   1      1    Fm3
.....................................................................aagcaccagguuauauca........................   4      0    Fm3
.....................................................................aagcaccagguuauauAag.......................   2      1    Fm3
.....................................................................aagcaccagguuauaucag.......................   136    0    Fm3
.....................................................................aagcaccaggAuauaucag.......................   1      1    Fm3
.....................................................................aagcaccagguuauaucagU......................   3      1    Fm3
.....................................................................aagcaccagguuauauGagg......................   1      1    Fm3
.....................................................................aagcaccagguuauaucagg......................   80     0    Fm3
.....................................................................aagcaccagguuaCaucagg......................   1      1    Fm3
.....................................................................aagcaccagguuauauAagg......................   1      1    Fm3
.....................................................................aagcaccagguuauauUaggg.....................   3      1    Fm3
.....................................................................Nagcaccagguuauaucaggg.....................   1      1    Fm3
.....................................................................aagcaccaggCuauaucaggg.....................   2      1    Fm3
.....................................................................Uagcaccagguuauaucaggg.....................   2      1    Fm3
.....................................................................aagcaccagguuauaucUggg.....................   1      1    Fm3
.....................................................................aagcaccagCuuauaucaggg.....................   1      1    Fm3
.....................................................................aagcaccagguuauaucaggU.....................   2      1    Fm3
.....................................................................aagcaccGgguuauaucaggg.....................   1      1    Fm3
.....................................................................aagcaccagguuauaCcaggg.....................   1      1    Fm3
.....................................................................aagcaccagguuauaucCggg.....................   1      1    Fm3
.....................................................................aagcaccagguuauaucaggg.....................   455    0    Fm3
.....................................................................aagcaccaggAuauaucaggg.....................   2      1    Fm3
.....................................................................aagcaccagguuauauGaggg.....................   1      1    Fm3
.....................................................................aagAaccagguuauaucagggu....................   4      1    Fm3
.....................................................................aagcaccaAguuauaucagggu....................   1      1    Fm3
.....................................................................aagcaUcagguuauaucagggu....................   8      1    Fm3
.....................................................................aagcaccagguuauaucagggA....................   8      1    Fm3
.....................................................................aagcaccGgguuauaucagggu....................   9      1    Fm3
.....................................................................Nagcaccagguuauaucagggu....................   12     1    Fm3
.....................................................................aCgcaccagguuauaucagggu....................   2      1    Fm3
.....................................................................aagcaccagguuauauUagggu....................   32     1    Fm3
.....................................................................aagcaccagguuauaucagAgu....................   1      1    Fm3
.....................................................................aagcaccagguuauaucagCgu....................   1      1    Fm3
.....................................................................aagcaccagguuauaucagggu....................   13074  0    Fm3
.....................................................................aagcaccagguuauGucagggu....................   5      1    Fm3
.....................................................................aagcaccagguuaAaucagggu....................   1      1    Fm3
.....................................................................aagcaccagguuauaucUgggu....................   43     1    Fm3
.....................................................................aagcaccagguuauaucaggAu....................   2      1    Fm3
.....................................................................aagcaccaggGuauaucagggu....................   18     1    Fm3
.....................................................................aaCcaccagguuauaucagggu....................   1      1    Fm3
.....................................................................aagcCccagguuauaucagggu....................   1      1    Fm3
.....................................................................aaAcaccagguuauaucagggu....................   2      1    Fm3
.....................................................................aagcaccagguuCuaucagggu....................   1      1    Fm3
.....................................................................aagcaccaUguuauaucagggu....................   3      1    Fm3
.....................................................................aGgcaccagguuauaucagggu....................   4      1    Fm3
.....................................................................aagcGccagguuauaucagggu....................   3      1    Fm3
.....................................................................aagcaccagguuauaAcagggu....................   1      1    Fm3
.....................................................................Gagcaccagguuauaucagggu....................   2      1    Fm3
.....................................................................aagcaccagguuGuaucagggu....................   1      1    Fm3
.....................................................................aagcaccUgguuauaucagggu....................   4      1    Fm3
.....................................................................aagcaccagguuauaucGgggu....................   43     1    Fm3
.....................................................................aagcaccaggAuauaucagggu....................   26     1    Fm3
.....................................................................aaUcaccagguuauaucagggu....................   1      1    Fm3
.....................................................................Uagcaccagguuauaucagggu....................   9      1    Fm3
.....................................................................aagcaccagguuauaucCgggu....................   48     1    Fm3
.....................................................................aagcaccagguuauCucagggu....................   1      1    Fm3
.....................................................................aagcaccaggCuauaucagggu....................   13     1    Fm3
.....................................................................aagcacUagguuauaucagggu....................   6      1    Fm3
.....................................................................aagcaccagguuauauGagggu....................   21     1    Fm3
.....................................................................aagcaccagguuaCaucagggu....................   2      1    Fm3
```

Star **Mature**

```
cguuauaaucgagcuuaaugccuuacuugaacccugguauaucuugugugcuuuggcaguguuggaaccaagcaccagguuauaucagggugcuugcaucgcauguacaaa
.....................................................................aagcaccagAuuauaucagggu....................  3    1  Fm3
.....................................................................aagcaccagguuauaucagUgu....................  5    1  Fm3
.....................................................................aagcaccagguuUuaucagggu....................  1    1  Fm3
.....................................................................aagcaccagguuauUucagggu....................  1    1  Fm3
.....................................................................aagcaccagguuauaucaUggu....................  6    1  Fm3
.....................................................................aagcaccagguuauauAagggu....................  74   1  Fm3
.....................................................................aagcaccagguuauaucaAggu....................  1    1  Fm3
.....................................................................aagcacNagguuauaucagggu....................  1    1  Fm3
.....................................................................aUgcaccagguuauaucagggu....................  1    1  Fm3
.....................................................................aagcacAagguuauaucagggu....................  5    1  Fm3
.....................................................................aagcaccagguuauaucaggUu....................  1    1  Fm3
.....................................................................aagcaccagguuauaucagggC....................  101  1  Fm3
.....................................................................aagcaccagguuauaucaggguU...................  382  1  Fm3
.....................................................................aagcaccagguuauaucaggguA...................  85   1  Fm3
......................................................................agcaccagguuaAaucaggg.....................  1    1  Fm3
......................................................................agcaccagguuauaucaggg.....................  1    0  Fm3
......................................................................agcaccCgguuauaucagggu....................  1    1  Fm3
......................................................................agcaccagguAauaucagggu....................  1    1  Fm3
......................................................................agcaccagguuauaGcagggu....................  1    1  Fm3
......................................................................agcaccagguuauaucagggC....................  2    1  Fm3
......................................................................agcaccaggCuauaucagggu....................  1    1  Fm3
......................................................................agcaccaggGuauaucagggu....................  1    1  Fm3
......................................................................agcaccaggAuauaucagggu....................  1    1  Fm3
......................................................................agcaccagguuauaucaggUu....................  1    1  Fm3
......................................................................agcaccagguuauaucagggu....................  72   0  Fm3
......................................................................agcaccagguuauaAcagggu....................  1    1  Fm3
......................................................................agcaccagguuauaucaggguU...................  6    1  Fm3
```
